# Supplementary material for: Essential roles of the ANKRD31-REC114 interaction in meiotic recombination and mouse spermatogenesis
Source: bioRxiv. 2023 Jul 20:2023.04.27.538541. Originally published 2023 Apr 28. Preprint. [Version 2] doi: 10.1101/2023.04.27.538541 (PMC10168363; doi:10.1101/2023.04.27.538541)
Supplement: Supplement 2 [file media-2.pdf]

Supplemental information for:

Essential roles of the ANKRD31–REC114 interaction in meiotic recombination and mouse spermatogenesis

Jiaqi Xu, Tao Li, Soonjoung Kim, Michiel Boekhout, and Scott Keeney

This pdf contains:

Supplemental Figures S1-S3

Supplemental Tables S1-S3

Additional information, in separate .xls file:

Supplemental File 1

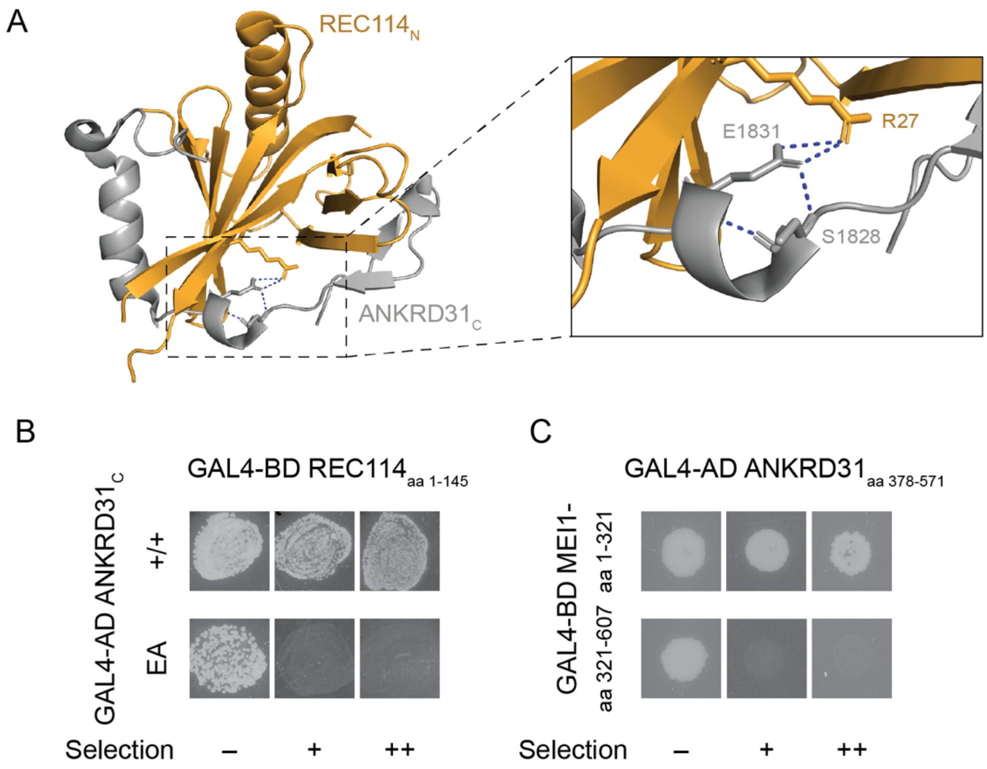

Figure S1. ANKRD31 interactions with REC114 and MEI1. (A) Crystal structure of ANKRD31$_C$ (gray) bound to the REC114$_N$ pleckstrin homology domain (yellow) (PDB: 6NXF) (Boekhout et al., 2019). The zoomed detail highlights contacts made by ANKRD31 Glu-1831. (B) Y2H interactions of REC114$_{aa\ 1\text{-}158}$ and wild type or EA mutant ANKRD31$_{C.}$ (C) Y2H interactions of full-length and fragments of ANKRD31 and MEI1. Cells express the indicated Gal4 activating domain (AD) and binding domain (BD) fusions. EV, empty vector. "Selection" indicates amino acid dropouts and aureobasidin to detect reporter activation at moderate (+) and high (++) stringency.

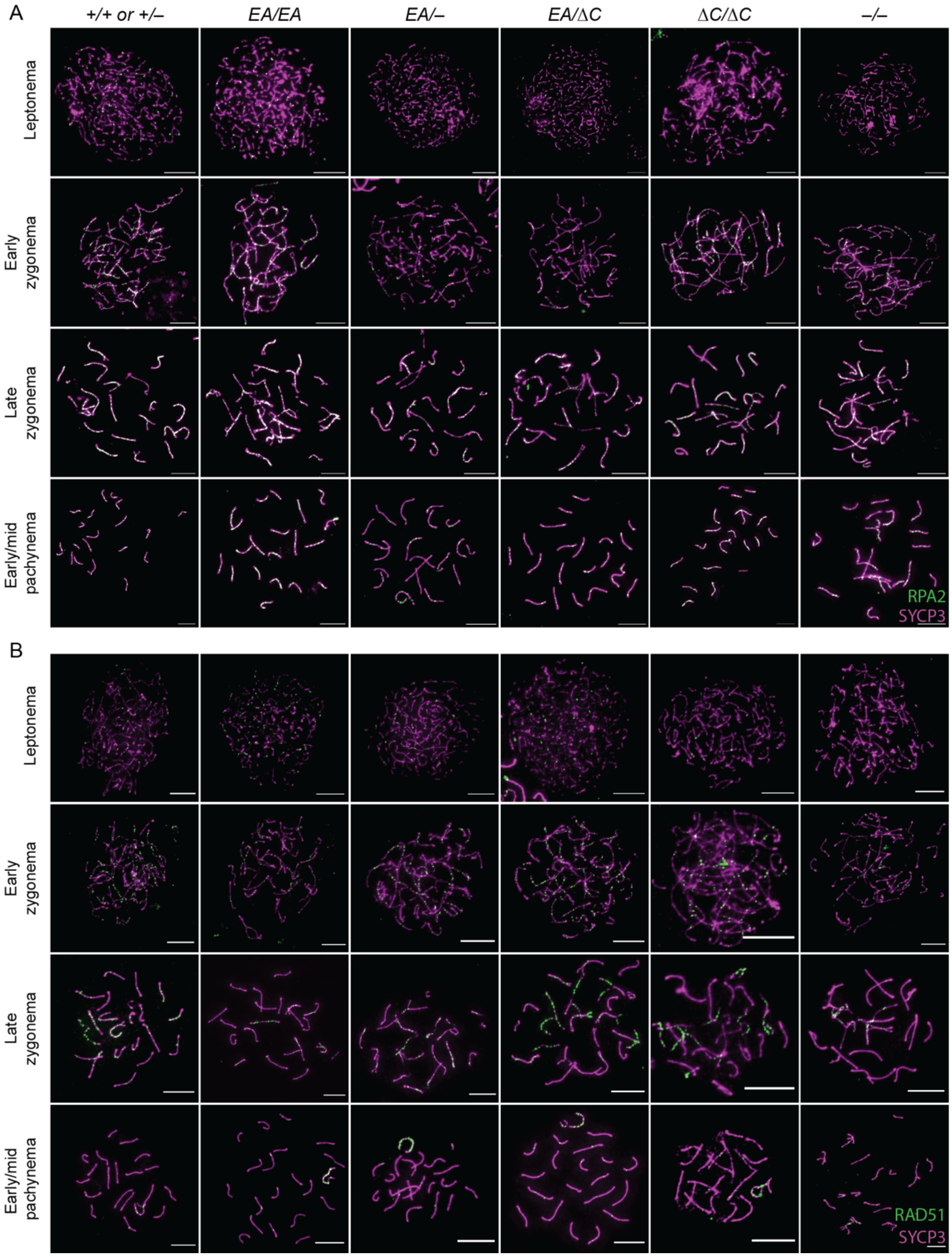

Figure S2. ANKRD31–REC114 interaction deficiencies cause progressive defects in DSB formation and recombination. Representative images of RPA2 staining (A) or RAD51 staining (B) of spermatocyte chromosome spreads. Quantification is presented in **Figure 7**. Scale bars, 10 μm.

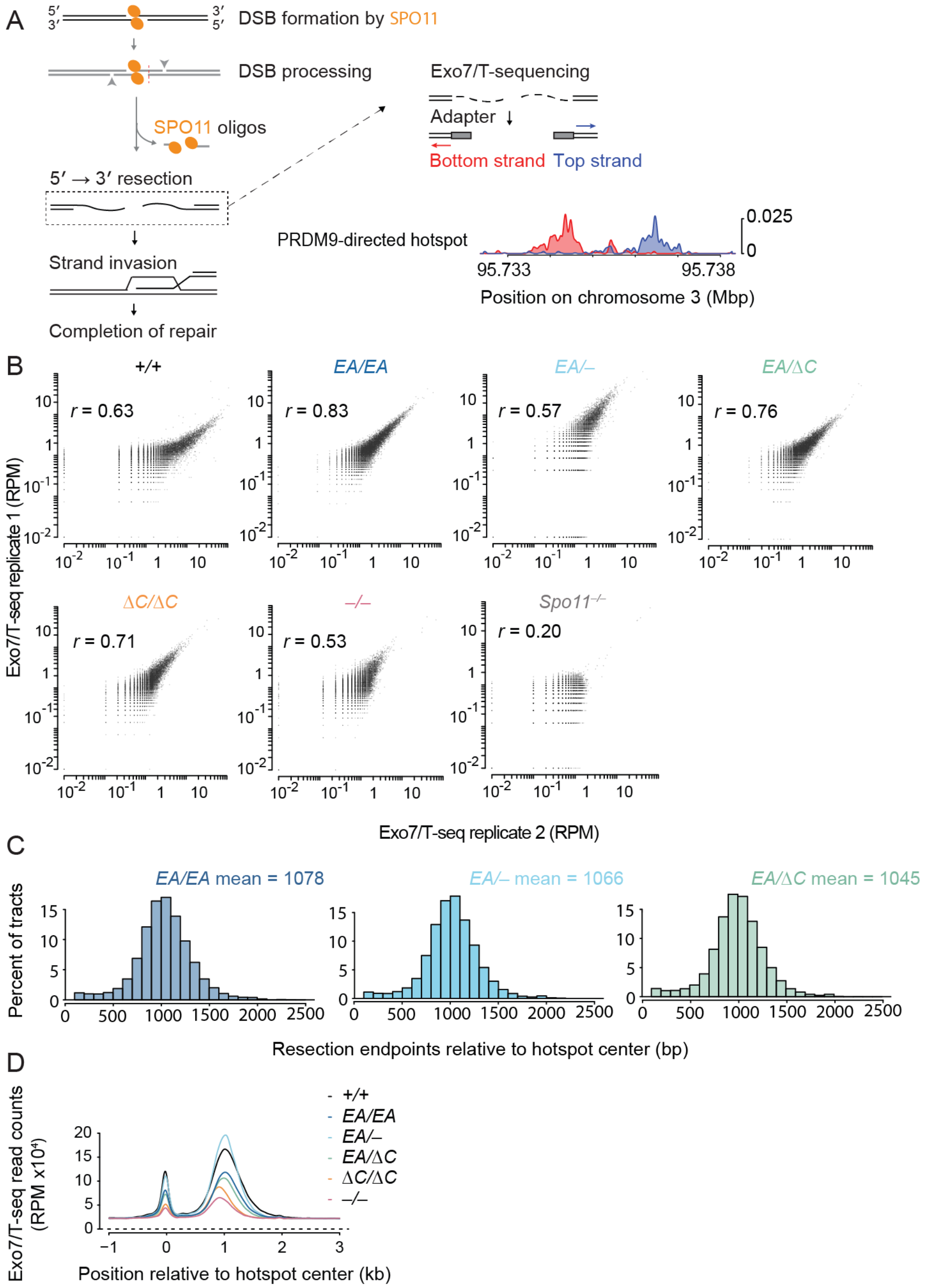

Figure S3. Schematic of Exo7/T sequencing and resection length. (A) Steps in DSB formation and processing and schematic of Exo7/T sequencing to detect resection endpoints. An example profile is shown from wild type at a PRDM9-directed hotspot in B6 mice. (B) Correlation (Pearson's *r*) of Exo7/T-seq read counts between biological replicates. Each point is a SPO11-oligo hotspot, with Exo7/T-seq signal summed from −2000 to −250 bp (bottom strand) and +250 to +2000 bp (top strand) around hotspot centers. Hotspots with ≤ 0 RPM were excluded for Pearson's *r* calculation. Hotspots with ≤ 10^{-2} RPM were set as 10^{-2} for plotting purposes only. Note that the correlation for the *Spo11*$^{-/-}$ replicates is poor because the sequencing signal represents mostly dispersed, nonspecific background. (C) Resection length distributions in

Ankrd31$^{EA/EA}$, *Ankrd31*$^{EA/–}$ and *Ankrd31*$^{EA/\Delta C}$. (D) Non-normalized, averaged Exo7/T-seq signals around PRDM9-directed hotspots (n = 13,960 SPO11-oligo hotspots in the C57BL/6J strain (33)). The bottom-strand reads were flipped and combined with the top-strand reads.

Supplemental Table S1. Overlap of Exo7/T-seq peak calls with SPO11-directed or default hotspots.

Overlap of Exo7/T-seq peak calls with SPO11-directed or default hotspots

	Total peak called	Merged peaks	Overlap with SPO11-directed hotspots	Overlap with default hotspots	Overlap with both	Overlap with neither
Ankrd31$^{+/+}$	5637	2704	2443	86	77	252
Ankrd31$^{EA/EA}$	6621	3213	3046	98	96	165
Ankrd31$^{EA/–}$	3743	2075	1975	59	57	98
Ankrd31$^{EA/\Delta C}$	5551	2931	2583	111	80	317
Ankrd31$^{\Delta C/\Delta C}$	6108	3562	1700	1432	99	529
Ankrd31$^{–/–}$	2899	1872	1187	400	41	326
Spo11$^{–/–}$	211	97	15	2	0	80

Supplemental Table S2. Primers for genotyping and cloning.

Primers for genotyping		
Genotype	Primer Name	Primer Sequence
Ankrd31^{EA}	18MGAnkF08	5’-CCGATCACGTATGCTCTGATACGG -3’
	18MGAnkR05	5’-ACTGACCATACGAGTTATAGTGCAGG -3’
Ankrd31$^{\Delta C}$	18MGAnkF05	5’-AGCAGGTACTTCCAGAGAGTCGAT -3’
	18MGAnkR03	5’-GCTAACTAAGCTACCAAGAAAGCAGAGC -3’

pGAD-ANKRD31 cloning primers		
Ankrd31^{EA}		5’-CTTCAAATCAGCAAGCAGGCGC-3’
		5’-CATTACATGGCATGGCAGAAGCGCC-3’
Ankrd31$^{\Delta C}$		5’-CAGAAGCAGGAGCTTCTGCCATGCCATGTAATGGAGCAG-3’
		5’-AGAAGCTCCTGCTTCTGATTTGAAGTATCTCTTTTATCTGT-3’

Supplemental Table S3. Antibodies.

Primary Antibodies

Target	Dilution for IF	Dilution for immunoblot	Host species	Supplier	Catalog number
γH2AX	1:6000	n/a	Rabbit	Abcam	ab2893
DMC1 (H100)	1:100	n/a	Rabbit	Santa Cruz	sc-22768
MLH1	1:25	n/a	Mouse	BD-Pharmingen	51-1327GR
RPA2	1:1000	n/a	Rabbit	Abcam	ab76420
RAD51	1:100	n/a	Rabbit	Aviva	ARP33450
SYCP3 (D-1)	1:300	n/a	Mouse	Santa Cruz	sc-74569
SYCP3	1:200	n/a	Rabbit	Nova	NB300-232
SYCP1	1:200	n/a	Rabbit	Abcam	ab15090
REC114	1:200	n/a	Rabbit	Stanzione et al., 2016	n/a
ANKRD31	1:200	1:4000	Guinea Pig	Boekhout et al., 2019	n/a

Secondary Antibodies

Target	Dilution for IF	Fluorophore	Host species	Supplier	Catalog number
anti Guinea pig	1:400	Alexa-488	Goat	Mol Probes	A-11073
anti Mouse	1:400	Alexa-594	Goat	Mol Probes	A-11005
anti Mouse	1:400	Alexa-594	Donkey	Life Technologies	A-21203
anti Mouse	1:400	Alexa-488	Donkey	Life Technologies	A-21202
anti Rabbit	1:400	Alexa-594	Goat	Invitrogen	A-11012
anti Rabbit	1:400	Alexa-488	Goat	Invitrogen	A11034
anti Rabbit	1:400	Alexa-488	Donkey	Thermofisher Scientific	R37118
