# Supplementary material for: Essential roles of the ANKRD31-REC114 interaction in meiotic recombination and mouse spermatogenesis
Source: bioRxiv. 2023 Apr 28:2023.04.27.538541. Preprint. [Version 1] doi: 10.1101/2023.04.27.538541 (PMC10168363; doi:10.1101/2023.04.27.538541)
Supplement: Supplementary file 1 [file media-1.pdf]

Supplemental information for:

Essential roles of the ANKRD31–REC114 interaction in meiotic recombination and mouse spermatogenesis

Jiaqi Xu, Tao Li, Soonjoung Kim, Michiel Boekhout, and Scott Keeney

This pdf contains:

Supplementary Figures 1-3
Supplementary Tables 1 and 2

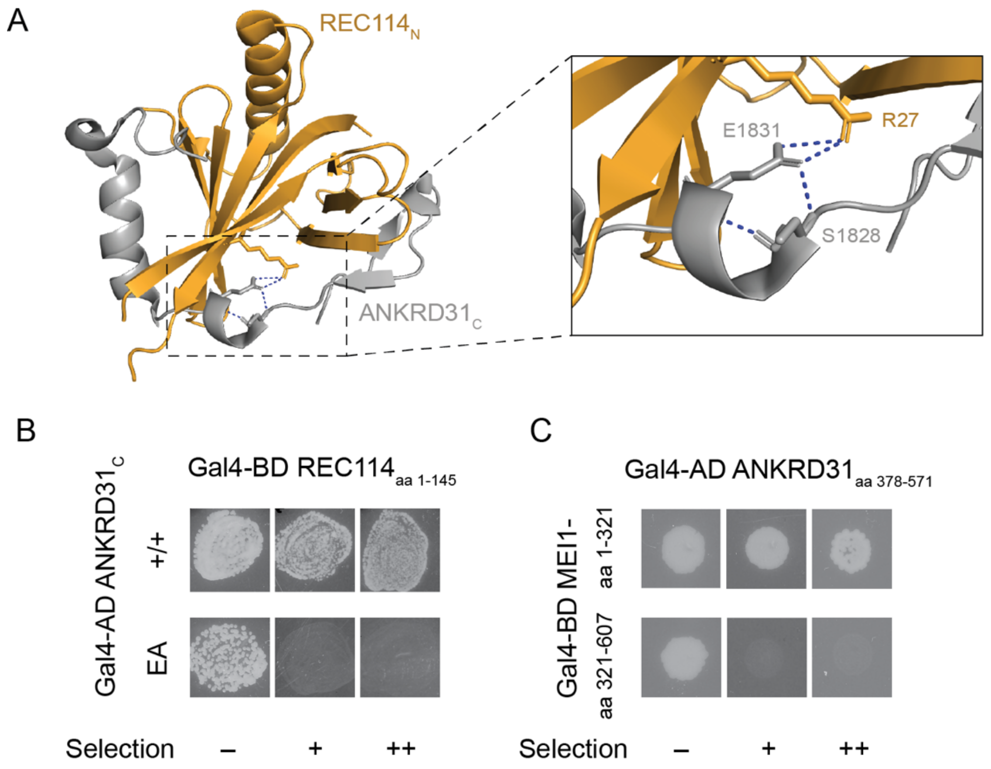

Figure S1. ANKRD31 interactions with REC114 and MEI1. (A) Crystal structure of $ANKRD31_C$ (gray) bound to the $REC114_N$ pleckstrin homology domain (yellow) (PDB: 6NXF) (Boekhout et al., 2019). The zoomed detail highlights contacts made by ANKRD31 Glu-1831. (B) Y2H interactions of $REC114_{aa\ 1\text{-}158}$ and wild type or E1831A mutant $ANKRD31_{C.}$ (C) Y2H interactions of full-length and fragments of ANKRD31 and MEI1. Cells express the indicated Gal4 activating domain (AD) and binding domain (BD) fusions. EV, empty vector. “Selection” indicates amino acid dropouts and aureobasidin to detect reporter activation at moderate (+) and high (++) stringency.

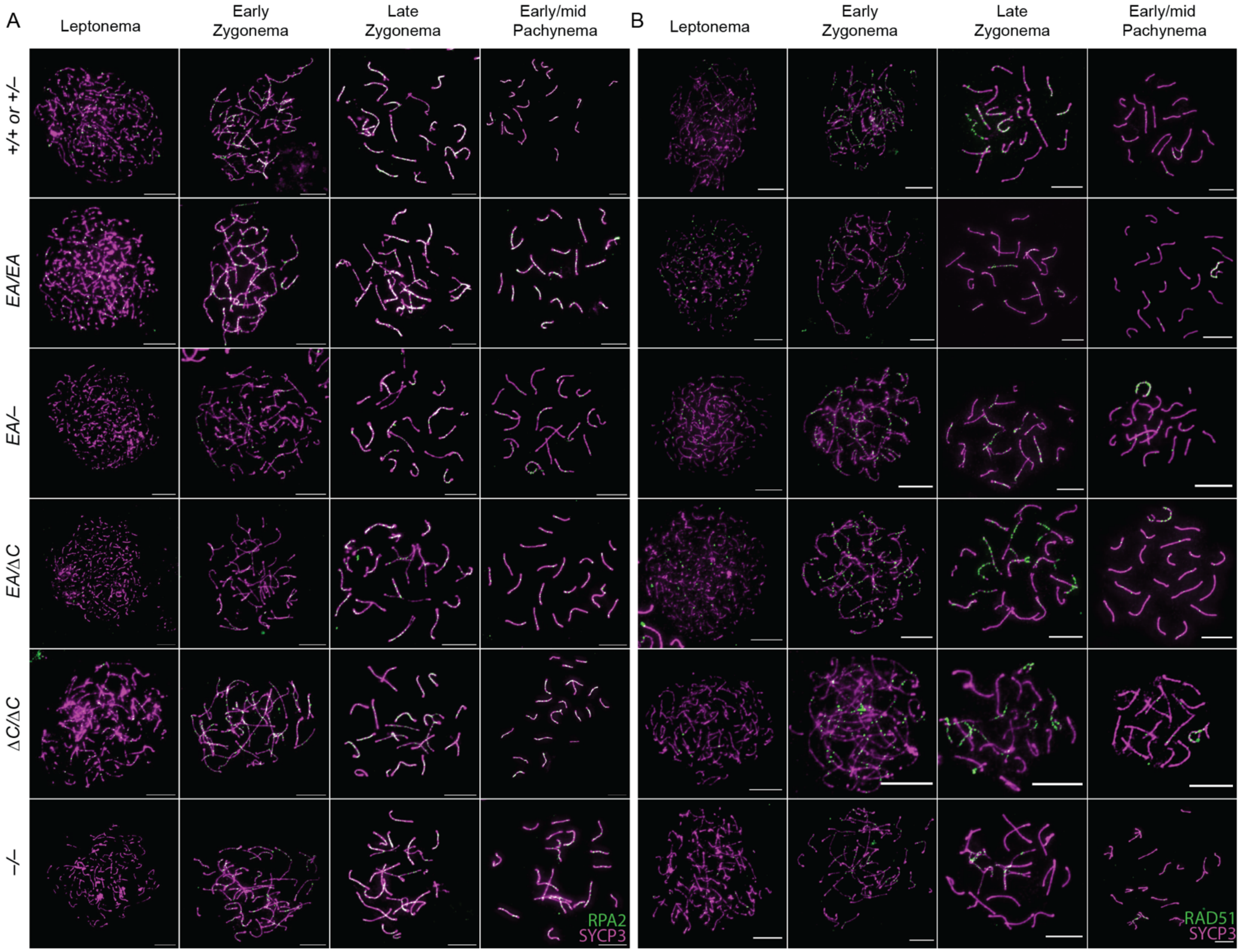

Figure S2. ANKRD31–REC114 interaction deficiencies cause progressive defects in DSB formation and recombination. Representative images of RPA2 staining (A) or RAD51 staining (B) of spermatocyte chromosome spreads. Quantification is presented in **Figure 7**. Scale bars, 10 µm.

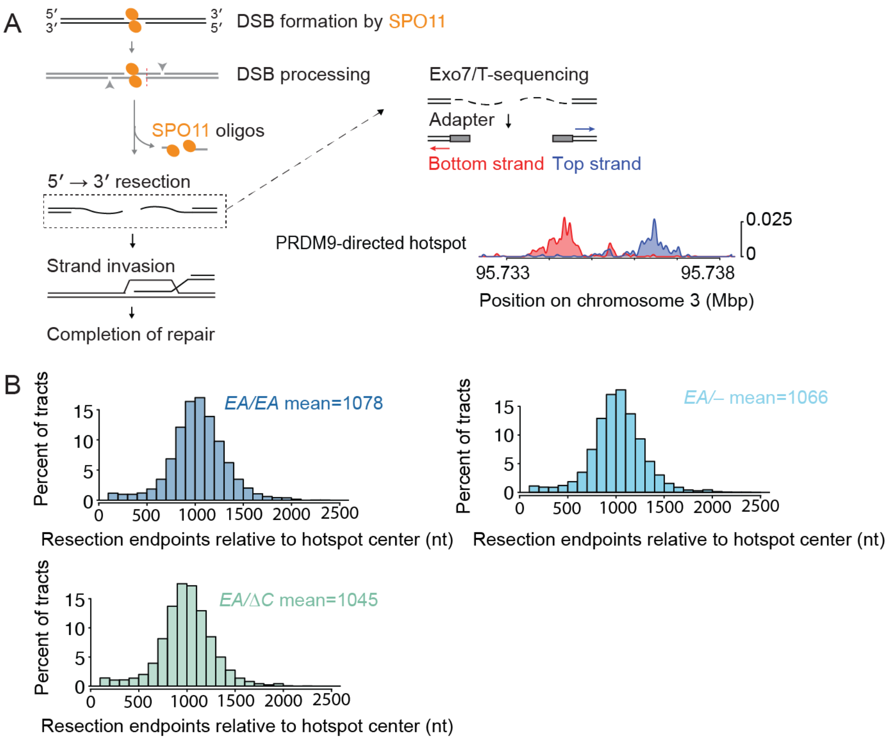

Figure S3. Schematic of Exo7/T sequencing and resection length. (A) Steps in DSB formation and processing and schematic of Exo7/T sequencing to detect resection endpoints. An example profile is shown from wild type at a PRDM9-directed hotspot in B6 mice. (B) Resection length distributions in $Ankrd31^{EA/EA}$, $Ankrd31^{EA/–}$ and $Ankrd31^{EA/\Delta C}$.

Supplemental Table S1. Primers for genotyping and cloning.

Primers for genotyping

Genotype	Primer Name	Primer Sequence
Ankrd31^{EA}	18MGAnkF08	5'-CCGATCACGTATGCTCTGATACGG -3'
	18MGAnkR05	5'-ACTGACCATACGAGTTATAGTGCAGG -3'
Ankrd31$^{\Delta C}$	18MGAnkF05	5'-AGCAGGTACTTCCAGAGAGTCGAT -3'
	18MGAnkR03	5'-GCTAACTAAGCTACCAAGAAAGCAGAGC -3'

pGAD-ANKRD31 cloning primers

Ankrd31^{EA}		5'-CTTCAAATCAGCAAGCAGGCGC-3'
		5'-CATTACATGGCATGGCAGAAGCGCC-3'
Ankrd31$^{\Delta C}$		5'-CAGAAGCAGGAGCTTCTGCCATGCCATGTAATGGAGCAG-3'
		5'-AGAAGCTCCTGCTTCTGATTTGAAGTATCTCTTTTATCTGT-3'

Supplemental Table S2. Antibodies.

Primary Antibodies

Target	Dilution for IF	Dilution for immunoblot	Host species	Supplier	Catalog number
γH2AX	1:6000	n/a	Rabbit	Abcam	ab2893
DMC1 (H100)	1:100	n/a	Rabbit	Santa Cruz	sc-22768
MLH1	1:25	n/a	Mouse	BD-Pharmingen	51-1327GR
RPA2	1:1000	n/a	Rabbit	Abcam	ab76420
RAD51	1:100	n/a	Rabbit	Aviva	ARP33450
SYCP3 (D-1)	1:300	n/a	Mouse	Santa Cruz	sc-74569
SYCP3	1:200	n/a	Rabbit	Nova	NB300-232
SYCP1	1:200	n/a	Rabbit	Abcam	ab15090
REC114	1:200	n/a	Rabbit	Stanzione et al., 2016	n/a
ANKRD31	1:200	1:4000	Guinea Pig	Boekhout et al., 2019	n/a

Secondary Antibodies

Target	Dilution for IF	Fluorophore	Host species	Supplier	Catalog number
anti Guinea pig	1:400	Alexa-488	Goat	Mol Probes	A-11073
anti Mouse	1:400	Alexa-594	Goat	Mol Probes	A-11005
anti Mouse	1:400	Alexa-594	Donkey	Life Technologies	A-21203
anti Mouse	1:400	Alexa-488	Donkey	Life Technologies	A-21202
anti Rabbit	1:400	Alexa-594	Goat	Invitrogen	A-11012
anti Rabbit	1:400	Alexa-488	Goat	Invitrogen	A11034
anti Rabbit	1:400	Alexa-488	Donkey	Thermofisher Scientific	R37118
